# Mortality in Patients with HIV-1 Infection Starting Antiretroviral Therapy in South Africa, Europe, or North America: A Collaborative Analysis of Prospective Studies

**DOI:** 10.1371/journal.pmed.1001718

**Published:** 2014-09-09

**Authors:** Andrew Boulle, Michael Schomaker, Margaret T. May, Robert S. Hogg, Bryan E. Shepherd, Susana Monge, Olivia Keiser, Fiona C. Lampe, Janet Giddy, James Ndirangu, Daniela Garone, Matthew Fox, Suzanne M. Ingle, Peter Reiss, Francois Dabis, Dominique Costagliola, Antonella Castagna, Kathrin Ehren, Colin Campbell, M. John Gill, Michael Saag, Amy C. Justice, Jodie Guest, Heidi M. Crane, Matthias Egger, Jonathan A. C. Sterne

**Affiliations:** 1 Centre for Infectious Disease Epidemiology and Research, School of Public Health and Family Medicine, Faculty of Health Sciences, University of Cape Town, Cape Town, South Africa; 2 Department of Health, Provincial Government of the Western Cape, Cape Town, South Africa; 3 School of Social and Community Medicine, University of Bristol, Bristol, United Kingdom; 4 Division of Epidemiology and Population Health, British Columbia Centre for Excellence in HIV/AIDS, Vancouver, Canada; 5 Faculty of Health Sciences, Simon Fraser University, Burnaby, Canada; 6 Department of Biostatistics, Vanderbilt University School of Medicine, Nashville, Tennessee, United States of America; 7 Centro Nacional de Epidemiología, Instituto de Salud Carlos III, Madrid, Spain; 8 University of Bern, Institute for Social and Preventive Medicine, Bern, Switzerland; 9 Research Department of Infection and Population Health, UCL Medical School, London, United Kingdom; 10 McCord Hospital, Durban, South Africa; 11 Africa Centre for Health and Population Studies, University of KwaZulu-Natal, Somkhele, South Africa; 12 Médecins Sans Frontières, Khayelitsha, South Africa; 13 Center for Global Health and Development, Boston University, Boston, Massachusetts, United States of America; 14 Stichting HIV Monitoring, Amsterdam, The Netherlands; 15 Department of Global Health and Division of Infectious Diseases, Academic Medical Center, University of Amsterdam, and Amsterdam Institute for Global health and Development, Amsterdam, the Netherlands; 16 INSERM, Centre INSERM U897 “Epidémiologie et Biostatistique”, Bordeaux, France; 17 Université Bordeaux, Institut de Santé Publique Epidémiologie Développement (ISPED), Bordeaux, France; 18 Sorbonne Universités, UPMC Univ Paris 06, UMR_S 1136, Institut Pierre Louis d'Epidémiologie et de Santé Publique, Paris, France; 19 INSERM, UMR_S 1136, Institut Pierre Louis d'Epidémiologie et de Santé Publique, Paris, France; 20 Infectious Diseases Department, San Raffaele Scientific Institute, Milan, Italy; 21 First Department of Internal Medicine, University Hospital of Cologne, Germany; 22 Centre d'Estudis Epidemiològics sobre les Infeccions de Transmissió Sexual i Sida de Catalunya (CEEISCAT), Institut català d'Oncologia (ICO), Agència Salut Pública de Catalunya (ASPC), Generalitat de Catalunya, Badalona, Spain; 23 CIBER Epidemiología y Salud Pública (CIBERESP), Madrid, Spain; 24 Division of Infectious Diseases, University of Calgary, Calgary, Canada; 25 Division of Infectious Disease, Department of Medicine, University of Alabama, Birmingham, Alabama, United States of America; 26 Yale University School of Medicine, New Haven, Connecticut, United States of America; 27 VA Connecticut Healthcare System, West Haven, Connecticut, United States of America; 28 HIV Atlanta VA Cohort Study (HAVACS), Atlanta Veterans Affairs Medical Center, Decatur, Georgia, United States of America; 29 Center for AIDS Research, University of Washington, Seattle, Washington, United States of America; Rwanda Ministry of Health, Rwanda

## Abstract

**Background:**

High early mortality in patients with HIV-1 starting antiretroviral therapy (ART) in sub-Saharan Africa, compared to Europe and North America, is well documented. Longer-term comparisons between settings have been limited by poor ascertainment of mortality in high burden African settings. This study aimed to compare mortality up to four years on ART between South Africa, Europe, and North America.

**Methods and Findings:**

Data from four South African cohorts in which patients lost to follow-up (LTF) could be linked to the national population register to determine vital status were combined with data from Europe and North America. Cumulative mortality, crude and adjusted (for characteristics at ART initiation) mortality rate ratios (relative to South Africa), and predicted mortality rates were described by region at 0–3, 3–6, 6–12, 12–24, and 24–48 months on ART for the period 2001–2010. Of the adults included (30,467 [South Africa], 29,727 [Europe], and 7,160 [North America]), 20,306 (67%), 9,961 (34%), and 824 (12%) were women. Patients began treatment with markedly more advanced disease in South Africa (median CD4 count 102, 213, and 172 cells/µl in South Africa, Europe, and North America, respectively). High early mortality after starting ART in South Africa occurred mainly in patients starting ART with CD4 count <50 cells/µl. Cumulative mortality at 4 years was 16.6%, 4.7%, and 15.3% in South Africa, Europe, and North America, respectively. Mortality was initially much lower in Europe and North America than South Africa, but the differences were reduced or reversed (North America) at longer durations on ART (adjusted rate ratios 0.46, 95% CI 0.37–0.58, and 1.62, 95% CI 1.27–2.05 between 24 and 48 months on ART comparing Europe and North America to South Africa). While bias due to under-ascertainment of mortality was minimised through death registry linkage, residual bias could still be present due to differing approaches to and frequency of linkage.

**Conclusions:**

After accounting for under-ascertainment of mortality, with increasing duration on ART, the mortality rate on HIV treatment in South Africa declines to levels comparable to or below those described in participating North American cohorts, while substantially narrowing the differential with the European cohorts.

*Please see later in the article for the Editors' Summary*

## Abstract 2

Analyzing survival in HIV treatment cohorts, Andrew Boulle and colleagues find mortality rates in South Africa comparable to or better than those in North America by 4 years after starting antiretroviral therapy.

*Please see later in the article for the Editors' Summary*

## Editors' Summary

**Background:**

AIDS has killed about 36 million people since the first recorded case of the disease in 1981, and a similar number of people (including 25 million living in sub-Saharan Africa) are currently infected with HIV, the virus that causes AIDS. HIV destroys immune system cells (including CD4 cells, a type of lymphocyte), leaving infected individuals susceptible to other serious infections. Early in the AIDS epidemic, HIV-positive people usually died within 10 years of becoming infected. In 1996, effective antiretroviral therapy (ART) became available and, for people living in high-income countries, HIV infection became a chronic condition. But ART was expensive, so HIV/AIDS remained largely untreated and fatal in resource-limited countries. Then, in 2003, the international community began to work towards achieving universal access to ART. By the end of 2012, nearly two-thirds of HIV-positive people (nearly 10 million individuals) living in low- and middle-income countries who were eligible for treatment because their CD4 cell count had fallen below 350/mm^3^ blood or because they had developed an AIDS-defining condition were receiving treatment.

**Why Was This Study Done?:**

It is known that a larger proportion of HIV-positive patients starting ART die during the first year of treatment in sub-Saharan Africa than in Europe and North America. This difference arises in part because patients in resource-limited settings tend to have lower CD4 counts when they start treatment than patients in wealthy countries. However, the lack of reliable data on mortality (death) in resource-limited settings has made it hard to compare longer-term outcomes in different settings. Information on the long-term outcomes of HIV-positive patients receiving ART in resource-limited countries is needed to guide the development of appropriate health systems and treatment regimens in these settings. In this collaborative analysis of prospective cohort studies, the researchers compare mortality up to 4 years on ART in South Africa, Europe, and North America. A prospective cohort study follows a group of individuals over time to see whether differences in specific characteristics at the start of the study affect subsequent outcomes. A collaborative analysis combines individual patient data from several studies.

**What Did the Researchers Do and Find?:**

The researchers combined data from four South Africa cohorts of HIV-positive patients starting ART included in the International Epidemiologic Databases to Evaluate AIDS South African (IeDEA-SA) collaboration with data from six North American cohorts and nine European cohorts included in the ART Cohort Collaboration (ART-CC). The South African cohorts were chosen because unusually for studies undertaken in countries in sub-Saharan Africa the vital status of patients (whether they had died) who had been lost to follow-up in these cohorts could be obtained from the national population register. Patients in South Africa began treatment with more advanced disease (indicated by a lower average CD4 count) than patients in Europe or North America. Notably, high early mortality after starting ART in South Africa occurred mainly in patients starting ART with a CD4 count below 50 cells/mm^3^. The cumulative mortality after 4 years of ART was 16.6%, 4.7%, and 15.3% in South Africa, Europe, and North America, respectively. After adjusting for patient characteristics at ART initiation, the mortality rate among patients beginning ART was initially lower in Europe and North American than in South Africa. However, although the adjusted mortality rate in Europe remained lower than the rate in South Africa, the rate in North America was higher than that in South Africa between 24 and 48 months on ART.

**What Do These Findings Mean?:**

Although the linkage to national vital registration systems (databases of births and deaths) undertaken in this collaborative analysis is likely to have greatly reduced bias due to under-ascertainment of mortality, the accuracy of these findings may still be limited by differences in how this linkage was undertaken in different settings. Nevertheless, these findings suggest that mortality among HIV-infected patients receiving ART in South Africa, although initially higher than in Europe and North America, rapidly declines with increasing duration on ART and, after 4 years of treatment, approaches the rate seen in high-income settings. Intriguingly, these findings also highlight the relatively higher late mortality in North America compared to either Europe or South Africa, a result that needs to be investigated to explore the extent to which differences in mortality ascertainment, patient characteristics and comorbidities, or health systems and treatment regimens contribute to variations in outcomes among HIV-positive patients in various settings.

**Additional Information:**

Please access these websites via the online version of this summary at http://dx.doi.org/10.1371/journal.pmed.1001718.

- This study is further discussed in a *PLOS Medicine* Perspective by Agnes Binagwaho and colleagues
- Information is available from the US National Institute of Allergy and Infectious Diseases on HIV infection and AIDS
- NAM/aidsmap provides basic information about HIV/AIDS, and summaries of recent research findings on HIV care and treatment
- Information is available from Avert, an international AIDS charity, on many aspects of HIV/AIDS, including information on universal access to ART, on HIV and AIDS in sub-Saharan Africa, and on HIV and AIDS in South Africa (in English and Spanish)
- The World Health Organization provides information on all aspects of HIV/AIDS (in several languages); its 2013 Consolidated guidelines on the use of antiretroviral drugs for treating and preventing HIV infections: recommendations for a public health approach are available
- The 2013 UNAIDS World AIDS Day Report provides up-to-date information about the AIDS epidemic and efforts to halt it
- Information about the International Epidemiologic Databases to Evaluate AIDS South African (IeDEA-SA) collaboration and about the ART Cohort Collaboration is available
- Personal stories about living with HIV/AIDS are available through Avert, Nam/aidsmap, and Healthtalkonline

## Introduction

Antiretroviral therapy (ART) for HIV-infected patients has been routinely available in some Sub-Saharan African settings for more than a decade. Early analyses of treatment cohorts in this region focussed on demonstrating the feasibility and subsequently the scalability of ART provision in high-burden but resource-constrained settings [1]–[5]. In South Africa alone more than 2 million people have been initiated on ART, and there is emerging evidence of substantial population-level declines in mortality [6].

Most data on long-term prognosis of patients started on ART continue to be derived from Europe and North America [7], in part because of concerns about mortality ascertainment in high burden settings [8]. Published comparisons between settings, and prognostic models from resource-limited settings, are limited to the first one or two years on ART [9]–[11]. Measured early mortality on ART has been higher in resource-limited settings including Southern Africa [9], with explanations for these differences focussing on demographic, socio-economic, biological, and health service factors [12]. Even in well-resourced settings there is substantial heterogeneity in mortality by region, individual cohorts, and according to patient characteristics [13],[14].

The availability in South Africa of an effective national vital registration system through a national population register (NPR) provides an opportunity to correct for under-ascertainment of mortality and to compare mortality between South Africa and other settings [15]. The aim of this analysis was to compare mortality up to four years on ART between cohorts from South Africa that were linked to the death registry, and cohorts from Europe and North America with near-complete ascertainment of deaths.

## Methods

### Ethics Statement

At all sites, institutional review boards (IRBs) had approved the collection of data and submission to the data centres at the Universities of Cape Town (IeDEA-SA) and Bristol (ART-CC). The IRBs at the universities of Cape Town and Bristol additionally provided respective approval for the IeDEA-SA and ART-CC collaboration activities under which this project was conducted. Both datasets were assembled in 2010.

### Settings and Cohorts

The South African cohorts form part of the International Epidemiologic Databases to Evaluate AIDS Southern African (IeDEA-SA) collaboration [16]. Data were restricted to the four cohorts, out of eight cohorts in South Africa, that were able to link to the NPR (Table S1). These cohorts include government primary-care clinics in Cape Town, hospital clinics in Johannesburg and Durban, and a rural program in KwaZulu-Natal Province. The ART Cohort Collaboration (ART-CC) brings together cohorts of treatment-naïve adult patients from Europe and North America [17]. Data were included from six North American and nine European cohorts (see collaborating centres).

### Patient Eligibility and Treatment Protocols

Eligible patients with HIV-1 were not previously exposed to ART and started ART between 2001 and 2009 aged 16 years and over. In South Africa adults were eligible for treatment if they had a CD4 count <200 cells/µl, or a WHO stage IV illness other than extra-pulmonary tuberculosis. Initial regimens included a nucleoside reverse-transcriptase inhibitor (NRTI) backbone of lamivudine with either zidovudine or stavudine (default from 2004), combined with a non-nuceloside reverse-transcriptase inhibitor (NNRTI), either nevirapine or efavirenz. Monitoring comprised six-monthly viral load and CD4 count testing. The cohorts in Europe and North America followed country-specific treatment guidelines, which generally comprised a first-line regimen of two NRTIs and either an NNRTI or boosted protease inhibitors. A wider range of NRTIs were accessible than in South Africa, including tenofovir and abacavir [18]. Monitoring of virologic and immunologic response was quarterly.

### Loss to Follow-up and Mortality Ascertainment

Loss to follow-up (LTF) was determined by closing the analysis for each cohort six months before database closure, and defining patients with no recorded death or visit in the window between analysis and database closure as being lost at their last visit. Patients with no outcome before analysis closure were censored at analysis closure.

The primary outcome was death from all causes. For each of the South African cohorts, civil identification numbers, where available, were crosschecked with the NPR prior to data transfer to confirm or ascertain dates of death. The European and North American cohorts recently described the frequency of death registry linkage as well as their own assessments of the completeness of mortality ascertainment [13]. All of the North American cohorts link to a population-based death registry at least annually, while only three of the European cohorts reported regular searching of a registry for unrecorded deaths (one cohort annually for patients <65 years old, and one each every two and three years, respectively). Estimates of completeness of mortality ascertainment ranged from 75% to 98%.

NPR data in South Africa were only able to distinguish natural from non-natural causes of death. Causes of death from most of the European and North American cohorts were coded using a procedure adapted from the CoDe protocol, [19],[20] and grouped as natural or non-natural, AIDS or non-AIDS, and infection-related or other.

### Statistical Methods

Patient characteristics at ART initiation (categorized or as medians with interquartile ranges) were described by region, including gender, age, CD4 count (within 6 months prior to initiation), viral load, mode of transmission (men who have sex with men, injection drug use, heterosexual, blood, other, or unknown), and year of ART initiation (Table 1). Although HIV in South Africa is predominantly heterosexually acquired [21],[22], data on mode of transmission were not available at an individual patient level for the South African patients. Clinical stage was characterised as advanced if WHO stage III or IV (South Africa) or CDC stage C (Europe and North America). Missing baseline values were multiply imputed by a chained equations approach and all resulting estimations were combined across five imputed datasets using Rubin's rules [23],[24]. The imputation model included baseline gender, CD4 count, viral load, year of ART initiation, HIV clinical stage, cohort, and region as well as the outcomes of duration of follow-up (continuous time) and mortality (see Textbox S1). The absence of data on hepatitis C (HCV) in South Africa precluded its inclusion in any combined analyses.

**Table 1:** **Cohort characteristics at ART initiation for three regions, 2001–2009.**

*Region*	South Africa		Europe		North America		Total	
*Patients included*	30,467		29,727		7,160		67,354	
**Gender, ** ***n*** ** (%)**								
Female	20,306	(66.6)	9,961	(33.5)	824	(11.5)	31,091	(46.2)
**Age (years)**								
Median (IQR)	35	(30–41)	38	(31–45)	43	(36–51)	37	(31–44)
**CD4 (cells/µl)**								
Observations, *n* (%)	27,211	(89.3)	29,727	(100.0)	7,160	(100.0)	64,098	(95.2)
<25	4,417	(16.2)	2,525	(8.5)	1,084	(15.1)	8,026	(12.5)
25–49	3,257	(12.0)	1,765	(5.9)	530	(7.4)	5,552	(8.7)
50–99	5,666	(20.8)	2,869	(9.7)	796	(11.1)	9,331	(14.6)
100–199	10,853	(39.9)	6,522	(21.9)	1,599	(22.3)	18,974	(29.6)
200–349	2,466	(9.1)	10,162	(34.2)	2,094	(29.2)	14,722	(23.0)
350–499	325	(1.2)	3,431	(11.5)	677	(9.5)	4,433	(6.9)
≥500	227	(0.8)	2,453	(8.3)	380	(5.3)	3,060	(4.8)
Median (IQR)	102	(42–166)	213	(103–316)	172	(60–281)	150	(61–246)
**Viral load (log_10_ copies/ml)**								
Observations, *n* (%)	12,129	(39.8)	29,724	(100.0)	7,160	(100.0)	49,013	(72.8)
<4	3,198	(26.4)	7,479	(25.2)	1,038	(14.5)	11,715	(23.9)
4–5	5,331	(44.0)	10,262	(34.5)	2,729	(38.1)	18,322	(37.4)
>5	3,600	(29.7)	11,983	(40.3)	3,393	(47.4)	18,976	(38.7)
Median (IQR)	4.6	(3.9–5.2)	4.8	(4.0–5.3)	5.0	(4.4–5.3)	4.8	(4.0–5.3)
**Year of ART initiation**								
2001–2003	1,022	(3.4%)	12,668	(42.6%)	3,606	(50.4%)	17,296	(25.7%)
2004–2006	16,221	(53.2%)	12,041	(40.5%)	2,671	(37.3%)	30,933	(45.9%)
2007–2009	13,224	(43.4%)	5,018	(16.9%)	883	(12.3%)	19,125	(28.4%)
**Clinical stage, ** ***n*** ** (%)**								
Observations	12,857	(42.2)	29,727	(100.0)	7,160	(100.0)	49,744	(73.9)
Advanced	10,278	(79.9)	6,401	(21.5)	2,012	(28.1)	18,691	(37.6)
**Mode of transmission, ** ***n*** ** (%)**								
MSM	—	—	9,539	(32.1)	1,030	(14.4)	10,569	(15.7)
IDU	—	—	2,563	(8.6)	1,626	(22.7)	4,189	(6.2)
Heterosexual	—	—	15,045	(50.6)	672	(9.4)	15,717	(23.3)
Blood	—	—	288	(1.0)	7	(0.1)	295	(0.4)
Other or unknown^a^	30,467	(100.0)	2,292	(7.7)	3,825	(53.4)	36,584	(54.3)
**HCV co-infected, ** ***n*** ** (%)**								
Observations	—	—	25,090	(84.4)	6,005	(83.9)	31,095	(46.2)
Infected	—	—	3,240	(12.9)	1,626	(27.1)	4,866	(15.6)

a Although HIV transmission in South Africa is predominantly heterosexual, individual level data on mode of transmission were not available.

Advanced stage refers to WHO stage IV or CDC Stage C; IDU, injecting drug users; IQR, inter-quartile range; MSM, men who have sex with men.

Among patients LTF in South Africa, those with civil identification numbers and therefore linkable to the NPR were up-weighted to represent all patients lost: within each cohort we took the inverse of the modelled probability (based on age, gender, CD4 count, year of ART initiation, and duration on ART when lost) of having a civil identifier as the weight for each linkable patient, in order to account for any differences between linkable and other patients LTF (Table S2). Patients not LTF received a weight of one, while those LTF and not linkable were given a weight of zero [25]. The weighted data for South Africa were combined with the data from Europe and North America for the Kaplan-Meier estimates and exponential models described below. Cumulative mortality up to four years on ART by region was estimated using a weighted Kaplan-Meier approach with bootstrapped (200) confidence intervals, for all patients and separately for patients with initial CD4 counts <50 and 50–199 cells/µl [25]. Mortality rates were described by region and individual cohort at 0–3, 3–6, 6–12, 12–24, and 24–48 months on ART. Shorter intervals were selected in the first year on ART due to the sharply declining mortality hazard soon after starting ART, and rapidly changing relative mortality comparing Europe and North America to South Africa. In addition to crude rates, to demonstrate inter-regional and inter-cohort heterogeneity in rates after adjustment, mortality rates were predicted within each time period after starting ART from an adjusted piecewise exponential parametric survival model for women aged 30–45 starting ART with a CD4 count 100–199 cells/µl, advanced clinical stage, viral load 4–5 log_10_ copies/ml, and starting ART in 2004–2007. The use of the exponential model simplified the prediction of rates for each duration interval on ART, while the implicit assumption of constant hazards within each interval produced almost identical inter-regional comparative mortality estimates when compared to piecewise spline-based flexible parametric models. Crude and similarly adjusted (for baseline gender, CD4 count, clinical stage, viral load, and calendar period) rate ratios were estimated at each duration for Europe and North America compared to South Africa (Table 2). Full outputs from these models together with the numbers of patients included and number of deaths are provided in Table 3. We performed several sensitivity analyses: restricted to particular patients (mode of HIV acquisition, calendar period of enrolment) or cohorts (self-reported mortality ascertainment level or frequency of registry linkage); upweighting the predicted regional mortality rates by the inverse of the weighted estimate of ascertainment completeness [13]; randomly allocating 20% of patients LTF in both Europe and North America to have died on the basis of a previous European study [26]; and differentially allocating 20% of patients LTF in Europe and 5% in North America to have died because of more complete checking of mortality registries in North America being likely to result in lower mortality in those remaining LTF.

**Table 2:** **Mortality rate ratios comparing Europe and North America to South Africa by duration on antiretroviral therapy, adjustment for baseline patient characteristics, and restricted to specific patients and cohorts in sensitivity analyses.**

Mortality Rate Ratio	Duration on ART (Months)				
	0–3	3–6	6–12	12–24	24–48
**Crude mortality rate ratio compared to South Africa (and 95% CI)**					
Europe	0.16 (0.14–0.19)	0.22 (0.18–0.26)	0.26 (0.22–0.31)	0.33 (0.28–0.38)	0.48 (0.40–0.57)
North America	0.29 (0.24–0.36)	0.50 (0.39–0.63)	0.76 (0.62–0.93)	1.10 (0.92–1.31)	2.21 (1.84–2.66)
**Adjusted for baseline patient characteristics** a					
Europe	0.30 (0.25–0.36)	0.41 (0.31–0.54)	0.39 (0.32–0.48)	0.37 (0.30–0.47)	0.46 (0.37–0.58)
North America	0.40 (0.31–0.50)	0.72 (0.53–0.97)	0.84 (0.66–1.07)	0.94 (0.74–1.20)	1.62 (1.27–2.05)
**Adjusted and corrected for cohort-reported under-ascertainment of mortality^b^**					
Europe	0.33 (0.22–0.49)	0.45 (0.28–0.74)	0.46 (0.30–0.70)	0.42 (0.27–0.66)	0.50 (0.32–0.77)
North America	0.39 (0.26–0.58)	0.71 (0.45–1.13)	0.81 (0.54–1.21)	0.99 (0.65–1.50)	1.54 (1.02–2.30)
**Adjusted and restricted to patients with sexual transmission in Europe and North America**					
Europe	0.28 (0.22–0.35)	0.37 (0.27–0.51)	0.37 (0.29–0.47)	0.33 (0.25–0.43)	0.46 (0.35–0.61)
North America	0.18 (0.10–0.35)	0.48 (0.25–0.89)	0.63 (0.39–1.02)	0.59 (0.37–0.93)	0.80 (0.50–1.28)
**Adjusted and restricted to cohorts with mortality ascertainment reported as >90%^c^**					
Europe	0.30 (0.23–0.39)	0.42 (0.30–0.60)	0.48 (0.37–0.64)	0.38 (0.29–0.51)	0.40 (0.29–0.54)
North America	0.41 (0.32–0.53)	0.72 (0.51–1.00)	0.95 (0.73–1.24)	0.88 (0.68–1.15)	1.56 (1.19–2.04)
**Adjusted and restricted to cohorts with regular death registry linkage^d^**					
Europe	0.36 (0.25–0.52)	0.39 (0.22–0.69)	0.27 (0.15–0.48)	0.49 (0.33–0.75)	0.66 (0.46–0.96)
North America	0.39 (0.30–0.50)	0.68 (0.50–0.92)	0.97 (0.73–1.28)	0.96 (0.74–1.26)	1.55 (1.18–2.04)
**Adjusted after assuming 20% of patients LTF subsequently died in Europe and North America**					
Europe	0.41 (0.35–0.48)	0.73 (0.59–0.90)	0.87 (0.73–1.04)	1.19 (1.00–1.40)	2.34 (1.96–2.80)
North America	0.48 (0.39–0.60)	0.96 (0.74–1.24)	1.25 (1.01–1.56)	1.72 (1.41–2.10)	3.59 (2.93–4.41)
**Adjusted after assuming 20% and 5% of patients LTF subsequently died in Europe and North America, respectively**					
Europe	0.39 (0.33–0.46)	0.74 (0.60–0.92)	0.86 (0.72–1.03)	1.22 (1.03–1.45)	2.55 (2.13–3.05)
North America	0.42 (0.33–0.52)	0.83 (0.64–1.09)	0.97 (0.76–1.23)	1.43 (1.16–1.77)	3.06 (2.49–3.78)
**Adjusted and restricted to patients with heterosexual transmission in Europe and North America**					
Europe	0.24 (0.18–0.31)	0.37 (0.26–0.53)	0.36 (0.27–0.48)	0.34 (0.24–0.47)	0.53 (0.39–0.71)
North America	0.13 (0.04–0.40)	0.84 (0.41–1.71)	0.84 (0.44–1.62)	0.54 (0.25–1.16)	1.21 (0.66–2.21)
**Adjusted and restricted to patients starting ART post-2004**					
Europe	0.24 (0.18–0.31)	0.41 (0.28–0.61)	0.38 (0.28–0.52)	0.30 (0.22–0.42)	0.43 (0.28–0.65)
North America	0.40 (0.28–0.56)	0.58 (0.36–0.92)	0.82 (0.57–1.18)	0.85 (0.59–1.21)	0.83 (0.47–1.44)
**Adjusted and restricted to patients starting ART post-2004 and in Europe and North America, heterosexual transmission**					
Europe	0.20 (0.14–0.28)	0.33 (0.20–0.55)	0.32 (0.21–0.50)	0.33 (0.22–0.51)	0.37 (0.22–0.63)
North America	0.09 (0.01–0.63)	0.22 (0.03–1.55)	0.60 (0.19–1.89)	0.19 (0.03–1.34)	1.69 (0.51–5.61)
**Adjusted and restricted to patients who achieved a viral load <400 copies/ml at 6 mo on ART**					
Europe			0.44 (0.32–0.59)	0.39 (0.29–0.53)	0.51 (0.36–0.71)
North America			0.74 (0.49–1.11)	0.82 (0.59–1.14)	1.38 (0.96–1.98)

a Adjustments were for baseline gender, CD4 count, clinical stage, viral load, and calendar period and are detailed in Table 3.

b Mortality rates were predicted from a multivariable model for each region and ART duration for a common group of patients (women aged 30–45 starting ART with a CD4 count 100–199 cells/µl, advanced clinical stage, viral load 4–5 log10 copies/ml, and starting ART in 2004–2007), and then corrected by the inverse of the weighted self-assessed completeness of mortality ascertainment for each region.

c Based on cohort-assessed completeness of mortality ascertainment and including seven European and six North American cohorts.

d All North American cohorts reported linking to death registries at least annually, whereas only three European cohorts provided linkage—one annually in patients <65 years old, one every two years, and one every three years.

**Table 3:** **Full multivariable models used to calculate mortality rate ratios comparing Europe and North America to South Africa by duration on antiretroviral therapy.**

Variables	Duration on ART (Months)				
	0–3	3–6	6–12	12–24	24–48
**Patients included (deaths^a^)**					
South Africa	30,467 (1431)	26,243 (609)	23,130 (585)	18,353 (513)	10,102 (251)
Europe	29,727 (237)	28,609 (146)	27,671 (184)	24,841 (235)	19,921 (268)
North America	7,160 (104)	6,863 (80)	6,655 (128)	5,928 (187)	4,506 (280)
**Gender**					
Female	0.80 (0.71–0.89)	0.86 (0.72–1.02)	0.83 (0.70–0.97)	0.74 (0.63–0.88)	0.79 (0.65–0.96)
**Age at ART initiation**					
<30 years	0.91 (0.80–1.05)	0.88 (0.72–1.09)	0.81 (0.65–1.00)	0.69 (0.55–0.86)	0.63 (0.49–0.82)
30–45 years	1.00 (Reference)	1.00 (Reference)	1.00 (Reference)	1.00 (Reference)	1.00 (Reference)
45–60 years	1.20 (1.05–1.38)	1.35 (1.11–1.63)	1.52 (1.28–1.80)	1.56 (1.32–1.84)	1.48 (1.25–1.74)
>60 years	2.39 (1.87–3.06)	2.31 (1.58–3.39)	2.00 (1.43–2.80)	2.77 (2.11–3.62)	2.55 (1.95–3.33)
**Year of ART initiation**					
2001–2003	1.00 (0.83–1.19)	0.96 (0.77–1.20)	1.06 (0.88–1.29)	1.08 (0.92–1.28)	1.31 (1.11–1.53)
2004–2006	1.00 (Reference)	1.00 (Reference)	1.00 (Reference)	1.00 (Reference)	1.00 (Reference)
2007–2009	1.23 (1.10–1.38)	1.33 (1.12–1.58)	0.99 (0.82–1.20)	0.89 (0.72–1.11)	0.53 (0.31–0.93)
**Baseline CD4 count (cells/µl)**					
0–24	3.40 (2.90–3.98)	3.05 (2.42–3.83)	2.40 (1.94–2.98)	1.67 (1.34–2.08)	1.26 (1.00–1.59)
25–49	2.55 (2.14–3.03)	2.07 (1.58–2.71)	1.82 (1.42–2.31)	1.38 (1.07–1.77)	1.15 (0.88–1.52)
50–99	1.51 (1.28–1.79)	1.63 (1.26–2.11)	1.52 (1.21–1.91)	1.19 (0.95–1.48)	1.17 (0.93–1.48)
100–199	1.00 (Reference)	1.00 (Reference)	1.00 (Reference)	1.00 (Reference)	1.00 (Reference)
200–349	0.77 (0.59–1.00)	0.95 (0.70–1.29)	0.88 (0.66–1.18)	0.78 (0.60–1.02)	0.71 (0.56–0.89)
350–500	0.40 (0.22–0.73)	0.52 (0.27–1.01)	0.49 (0.27–0.88)	0.43 (0.27–0.68)	0.75 (0.54–1.05)
**Baseline viral load (log10 copies/ml)**					
<4	0.98 (0.79–1.21)	1.11 (0.78–1.59)	1.02 (0.79–1.32)	1.08 (0.87–1.34)	1.03 (0.78–1.38)
4–5	1.00 (Reference)	1.00 (Reference)	1.00 (Reference)	1.00 (Reference)	1.00 (Reference)
>5	1.13 (0.90–1.41)	0.90 (0.73–1.10)	1.06 (0.87–1.28)	1.04 (0.81–1.34)	1.23 (1.02–1.49)
**Baseline clinical stage**					
Advanced stage	2.42 (1.83–3.21)	2.37 (1.66–3.37)	1.94 (1.59–2.37)	1.45 (1.19–1.78)	1.39 (1.17–1.65)
**Region**					
South Africa	1.00 (Reference)	1.00 (Reference)	1.00 (Reference)	1.00 (Reference)	1.00 (Reference)
Europe	0.30 (0.25–0.36)	0.41 (0.31–0.54)	0.39 (0.32–0.48)	0.37 (0.30–0.47)	0.46 (0.37–0.58)
North America	0.40 (0.31–0.50)	0.72 (0.53–0.97)	0.84 (0.66–1.07)	0.94 (0.74–1.20)	1.62 (1.27–2.05)

The above patient numbers and models are the basis for crude and adjusted mortality rate ratios as presented in Table 2 and Figure 3 and the predictions in Figure 4. The associations in these models are presented as adjusted rate ratios with corresponding 95% CIs in parentheses.

a Deaths in South Africa represent the estimated number of deaths after correction for mortality under-ascertainment through record linkage and re-weighting. The proportions of deaths that were documented prior to record linkage were 63%, 53%, 51%, 47%, and 43% for the successive durations on ART reflected in the table.

We determined the proportion of patients in each region who achieved virologic suppression at 6 months on ART (measured between 3 and 9 months). We conducted a sensitivity analysis in which mortality comparisons beyond 6 months on ART were restricted to patients who achieved virologic suppression at 6 months.

Background mortality was compared between regions using 1990 country mortality data proportional to cohort size [27], standardised by age and gender to the combined study population.

## Results

The final analysis included 67,354 patients, with 30,467 (45%), 29,727 (44%), and 7,160 (11%) patients from South Africa, Europe, and North America, respectively (Table 1), followed for a median of 1.6, 3.5, and 3.2 years prior to censoring at four years. Patients differed markedly between regions with respect to gender and mode of transmission. In South Africa 20,306 (67%) patients were women compared to 824 (12%) in North America. Acquisition of HIV was attributed to heterosexual contact in 15,045 (51%) patients in Europe and 672 (9%) in North America; data on mode of sexual transmission were not available for South African patients. Patients were younger in South Africa compared to Europe and North America (median 35, 38, and 43 years, respectively). The South African patients had more advanced disease at ART initiation, evidenced by lower CD4 counts (median 102 compared to 213 and 172 cells/µl in Europe and North America) and more advanced clinical stage. Almost half the South African patients (13,224, 43%) started ART in 2007 or later, in contrast to the other regions where ART was mostly started in earlier years.

In South Africa, 26,100 patients (85.7%) had known outcomes at analysis closure. Among the 4,367 (14.3%) patients LTF, 2,594 (57.4%) (Table S2) could be linked to the NPR, with 956 (36.9%) having died. A further 1,652 (5.4%) patients transferred care and were censored at the time of transfer. By comparison, 31% (Europe) and 18% (North America) of patients would have been similarly classified as LTF prior to analysis closure or four years of follow-up, while transfers were not distinguished from other losses to care in these cohorts.

**Figure 1:**
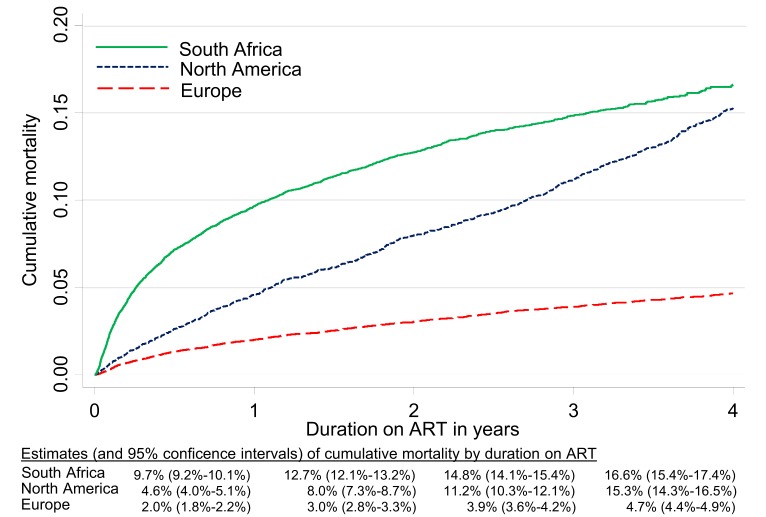
Cumulative incidence of mortality up to four years after start of ART by region, corrected in South Africa for mortality under-ascertainment.

**Figure 2:**
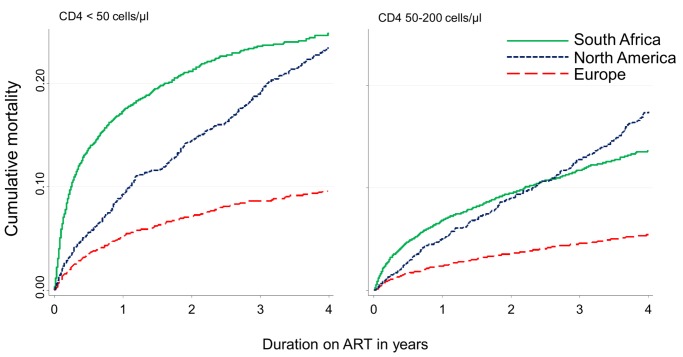
Cumulative incidence of mortality up to four years after start of ART by region and CD4 count at ART initiation, corrected in South Africa for mortality under-ascertainment.

The inclusion of NPR linkage data on vital status in South Africa more than doubled estimated cumulative all-cause mortality on ART at four years from 7.7% to 16.6% (95% CI 15.4%–17.4%). Crude cumulative four-year mortality was 4.7% (4.4%–4.9%) in Europe and 15.3% (14.3%–16.5%) in North America (Figure 1). Mortality after one year on ART was higher in South Africa (9.7%, 95% CI 9.2%–10.1%) than in Europe (2.0%, 1.8%–2.2%) or North America (4.6%, 4.0%–5.1%). The higher early mortality in South Africa was especially evident in patients with CD4 count <50 cells/µl at start of ART (Figure 2). For patients initiating ART with initial CD4 count between 50 and 199 cells/µl, crude cumulative all-cause mortality in North America at four years exceeded that in South Africa. In sensitivity analyses restricted in Europe and North America to patients with recorded heterosexually acquired HIV, crude cumulative mortality in North America was consistently lower than in the overall study population in South Africa and differences with Europe were reduced (Figures S1 and S2).

**Figure 3:**
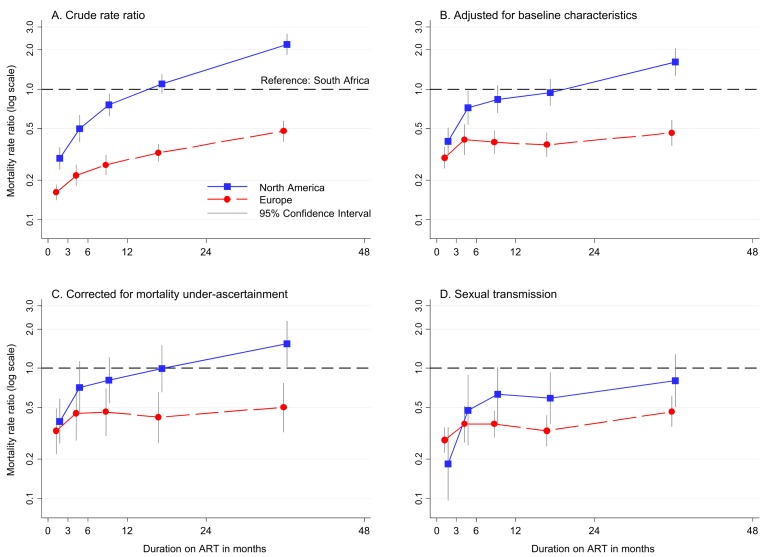
Relative mortality by region and duration on ART comparing European and North American cohorts to South Africa. (A) Crude rates, (B) adjusted for baseline covariates*, (C) corrected for cohort-assessed mortality under-ascertainment** and adjusted for baseline covariates, and (D) limited in Europe and North America to patients with sexual acquisition of HIV, adjusted for baseline covariates. *Adjusted for baseline gender, CD4 count, clinical stage, viral load, and calendar period. **In order to correct for cohort-assessed completeness of mortality ascertainment, mortality was predicted for each region and duration from a multivariable model for each cohort for women aged 30–45 starting ART with a CD4 count 100–199 cells/µl in 2004–2007, with advanced clinical stage and viral load 4–5 log copies/ml. Each mortality rate in Europe and North America was multiplied by the inverse of the cohort-assessed proportion of deaths ascertained (weighted estimate from participating cohorts), prior to converting to rate ratios relative to South Africa.

Figures 3A and 3B display crude and adjusted (for baseline characteristics) mortality rate ratios comparing Europe and North America with South Africa by duration on ART. Mortality rates were lower in Europe and North America than in South Africa during the first year of ART, lower in Europe but comparable in North America between 12 and 24 months, and lower in Europe and higher in North America between 24 and 48 months on ART (adjusted rate ratios [ARRs] 0.46, 95% CI 0.37–0.58 for Europe and 1.62, 95% CI 1.27–2.05 for North America, compared to South Africa) (Tables 2 and 3). This pattern was little changed by further correction for sites' self-estimated completeness of mortality ascertainment (Figure 3C). When analyses of mortality in Europe and North America were restricted to patients with sexual transmission, adjusted mortality rates were lower in Europe and North America than in South Africa for the first two years on ART, and lower in Europe but comparable to North America between 24 and 48 months (Figure 3D). These attenuated differences between Europe and North America were evident in additional sensitivity analyses with further restriction to patients in Europe and North America with recorded heterosexually acquired HIV (compared to the overall study population in South Africa), or those starting ART more recently (Table 2). Assuming that a proportion of patients LTF in European and North American cohorts had died resulted in higher mortality in both Europe and North America than in South Africa after 1 year on ART, while further attenuating differences between Europe and North America, especially when mortality in patients LTF was assumed to be lower in North America than in Europe.

**Figure 4:**
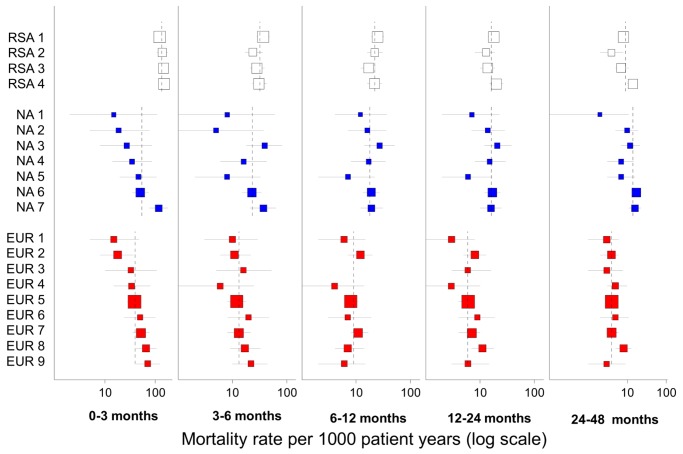
Predicted mortality by cohort and region. Predicted for women aged 30–45, starting ART with a CD4 count 100–199 cells/µl in 2004–2007, advanced clinical stage, and viral load 4–5 log copies/ml. The squares are scaled to the square root of cohort size, the horizontal lines represent 95% CIs, and the dotted vertical lines represent regional estimates for each duration. A mortality rate could not be estimated for one European cohort between 6 and 12 months on ART due to the absence of events. RSA, South Africa; NA, North America; EUR, Europe.

Viral load measurements at 6 months on ART were available for 55.6%, 86.7%, and 81.7% of patients in South Africa, Europe, and North America, respectively, with 89.5%, 89.5%, and 73.4%, respectively, achieving suppression to below 400 copies/ml. Adjusted comparisons of mortality restricted to patients who achieved suppression demonstrated broadly similar inter-regional differences to the primary adjusted comparison, but with a reduction in mortality rate ratios comparing North America to South Africa beyond a year on ART (Table 2). Predicted mortality by individual cohort was comparable for the South African cohorts up to two years on ART, but varied more substantially early on treatment between cohorts within Europe and North America (Figure 4).

The proportion of classifiable deaths that were classified as from unnatural causes was 2.8%, 5.1%, and 8.6% in South Africa, Europe, and North America, respectively. In Europe 525 (47.4%) of 1,107 coded deaths were due to AIDS compared to 128 (52.5%) of the 244 coded deaths in North America. In both of these regions 30% of deaths were due to infection, the majority being AIDS-defining (20%).

Standardised 1990 mortality from representative countries, illustrative of differences in mortality prior to the effects of HIV and hence of background mortality, was substantially lower in Europe and North America (41% and 46% of South Africa, respectively, 682 deaths/100,000 years in South Africa).

## Discussion

To our knowledge this is the first comparison of mortality up to four years after starting ART between high-income countries and a high HIV-burden setting in Southern Africa with near-complete mortality ascertainment. The high early mortality in patients in South Africa starting ART, which was first reported in an earlier comparative analysis [9], occurs mainly in patients who were severely immunosuppressed (CD4 count <50 cells/µl) at the start of ART. Differences in mortality rates between South Africa, Europe, and North America are markedly reduced or even reversed thereafter.

The ART cohort collaboration recently described substantial differences between mortality rates among HIV cohorts in Europe and North America [13], which arose from a combination of health services factors, patients' socio-economic and behavioural characteristics, co-morbidities such as HCV, and differential mortality ascertainment. The current analysis confirms that these differences are largely mediated through adverse outcomes in patients with transmission via injection drug use or with unknown mode of transmission given the attenuation in these differences in analyses restricted to patients with sexual transmission of HIV. ART treatment settings are often dichotomised as resource-limited and resource-replete, but the results reported here emphasise the importance of considering a range of contextual issues when comparing mortality between cohorts and settings, irrespective of region.

### Mortality Ascertainment

The manner in which LTF is incorporated into HIV cohort analyses can substantially impact findings [28],[29]. Analyses of South African ART data are increasingly based on a repeatable and structured method for incorporating data from the NPR [15],[25],[30],[31], which effectively ensures that every patient LTF is reclassified as alive or deceased. Half of the deaths in South Africa were ascertained based on death registry linkage, and it has been demonstrated for patients in the participating cohorts that between 90% and 95% of known deaths are identified by the registry [15]. The residual proportion of deaths not ascertained is therefore likely to be very low.

Most European and North American cohorts assume that patients LTF have the same outcomes as retained patients. Where mortality ascertainment through registries is frequent and comprehensive, as in the North American cohorts included here, this may over-estimate mortality because most deaths in patients LTF will already have been ascertained. If LTF is high without the possibility of registry linkage, as in many high burden settings [32],[33], or linkage is irregular or incomplete as in some European cohorts, mortality may conversely be underestimated. Correction for cohort-assessed completeness of mortality ascertainment had a limited effect on our results, possibly due to poor accuracy of this assessment. Assuming however that a plausible proportion of patients LTF in Europe and North America had died substantially reduced the differences between European and North American cohorts, particularly when mortality in these patients was assumed to be higher in Europe than North America in keeping with differences in the completeness and frequency of death registry linkage.

Future cohort analyses of mortality should be explicit as to the frequency and completeness of death registry linkage, the manner in which additional data from registries are incorporated into the data or analyses, and the analytic approach to defining and correcting for losses to care. Robust data on outcomes in patients LTF in Europe and North America will assist future analyses.

### Clinic Versus Population-Based Cohorts

Some of the South African and North American cohorts are geographically defined population-based cohorts. Mortality differences between clinic or hospital and population-based cohorts can be ascribed both to patient characteristics (population-based cohorts may include more marginalised patients with higher mortality) [13], and to biases resulting from patients moving from or being lost to facility-based care. Paradoxically higher on-ART mortality in a population-based cohort might reflect better coverage of hard-to-reach patients and lower population-level HIV-related mortality. Although in practice it may be problematic to strictly dichotomize cohorts as being clinic or population-based; potential differences in mortality ascertainment arising from service models requires consideration in on-ART mortality analyses.

### Access to and Quality of Health Care

High early mortality in South Africa relative to the other settings occurs mainly in patients with severe immunosuppression at ART initiation. Poor access to care is an important contributor to late presentation, whether due to disease burden outstripping public sector resources as in South Africa, or due to marginalised communities having limited access to health care in better resourced settings [34],[35].

There are likely pathogen- and disease-burden related contributions to higher early mortality on ART in South Africa, which are exacerbated in the context of delayed access to care, including undiagnosed tuberculosis, cryptococcal disease, and severe bacterial infections [12],[36]–[38]. Detailed data on causes of death were not available from the South African cohorts; however, published studies of both inpatient and outpatient ART mortality in South Africa suggest that the majority of deaths were due to AIDS-defining or HIV-associated associated infections, especially tuberculosis, which is frequently undiagnosed at the time of death [39],[40]. This finding contrasts with the 30% of classifiable deaths in Europe and North America that were infection-related.

It is also possible that measures used to adjust analyses for disease severity reflecting delayed access to care, such as CD4 count and clinical stage, may not fully capture differences in severity, resulting in residual confounding in comparative analyses.

In patients with advanced disease, background differences in health outcomes between regions, as reflected by differences in the pre-HIV-era mortality, may be accentuated in the early period on ART. By contrast it is possible that once well-established on ART, patients who have managed to access and remain part of a dedicated care program are advantaged relative to other public sector patients, narrowing differences that might be predicted based on comparisons of background mortality. This phenomenon has been described with respect to men and women in South Africa, where differentials in on-ART mortality are lower than for similarly aged citizens without HIV [30].

### HIV Disease Progression and Response to Treatment

Higher mortality in North America than South Africa after 2 years on ART appeared partially attributable to a lower proportion of patients achieving virologic suppression in North America, compared with South Africa and Europe. This finding is consistent with lower adherence to therapy in marginalised groups in some North American settings [41].

Although comparative data on disease progression were not available from South Africa, previous analyses comparing European and North American cohorts within the ART Cohort Collaboration demonstrated that inter-cohort differences in progression to AIDS are attenuated by the same factors that attenuate differences in mortality [13]. Interestingly, the ordering of cohorts by progression to AIDS differed from the ordering for mortality, which supports inter-cohort and inter-regional differences in mortality arising both from differences in the clinical progression of HIV and for other reasons.

### Patient Factors Independent of HIV Disease

Patients differ markedly between contexts, and this is crudely explored in our study by mode of HIV transmission. In some cohorts, particularly in the USA and Canada, many patients with HIV are from marginalised groups with high background mortality and prevalence of co-morbidities such as HCV. The importance of non-HIV mortality risk factors was demonstrated in the Danish HIV Cohort Study, where mothers of HIV-infected individuals were shown to have higher rates of myocardial infarction than population-matched controls [42], and non-HIV risk factors (co-morbidities including HCV and alcohol and drug abuse) explained a considerable component of differential mortality on treatment [43]. Co-morbidity may contribute to later mortality on ART in context-specific ways: for example HCV infection may impact later mortality in the North America cohorts in this study, in which a high proportion of patients are or were previously injecting drug users [44]. In South Africa where HIV affects the general population, those accessing care may have been exceptionally motivated citizens, especially in the early years of the programs when treatment availability was more limited.

### Strengths and Limitations

The analytical approach based on death registry linkage minimises bias resulting from under-ascertainment of mortality. The manner of incorporation of registry data differed, however, between the regions and could have still biased results, most importantly the less frequent linkages to mortality registries in Europe. The approach to accounting for patient transfers was also not standardized across regions. In the South African cohorts data on mode of HIV transmission, progression to AIDS, and cause of death were not available, and baseline data on viral load and clinical stage were available in less than half the patients, limiting the range and precision of comparative analyses. The four-year duration of follow-up is a further strength of the paper, but, given the continued rapid scaling up of ART and evolution of treatment guidelines, patterns of relative mortality may have evolved subsequent to the closure of this analysis. It was not possible however to use more recent data from South Africa, as following the initial linkage exercises, three of the included cohorts began incorporating the NPR mortality data into their primary data systems, invalidating the subsequent use of the up-weighting procedures described in this paper.

## Conclusions

With increasing duration on ART, mortality in HIV-infected patients on treatment in South Africa declines rapidly to levels approaching those in high-income settings. Contextual factors related to measurement, health services, and patient characteristics account for a large proportion of regional mortality variation and are key to interpreting mortality on ART both within and between settings. Such comparisons remain an invaluable tool for exploring treatment responses to the HIV pandemic and developing health systems that best serve patients in these different settings.

## Supporting Information

Figure S1 Cumulative incidence of mortality up to four years after start of ART by region, restricted in Europe and North America to patients with recorded heterosexually acquired HIV, corrected in South Africa for mortality under-ascertainment.
(EPS)

Figure S2 Cumulative incidence of mortality up to four years after start of ART by region and CD4 count at ART initiation, restricted in Europe and North America to patients with recorded heterosexually acquired HIV, corrected in South Africa for mortality under-ascertainment.
(EPS)

Table S1 Comparison at ART initiation of South African patients excluded from and included in the final analysis on the basis of cohort-level ability to link to the National Population Register.
(DOCX)

Table S2 Comparison at ART initiation of patients lost to follow-up in South Africa with and without civil identifiers enabling linkage to the population register.
(DOCX)

Textbox S1 Multiple Imputation of missing baseline values.
(DOCX)

## Abbreviations

ART: antiretroviral therapy
HCV: hepatitis C virus
LTF: lost to follow-up
NPR: national population register
