# Supplementary material for: Mortality in Patients with HIV-1 Infection Starting Antiretroviral Therapy in South Africa, Europe, or North America: A Collaborative Analysis of Prospective Studies
Source: PLoS Med. 2014 Sep 9;11(9):e1001718. doi: 10.1371/journal.pmed.1001718 (PMC4159124; doi:10.1371/journal.pmed.1001718)
Supplement: Table S1 — Comparison at ART initiation of South African patients excluded from and included in the final analysis on the basis of cohort-level ability to link to the National Population Register. (DOCX) [file pmed.1001718.s003.docx]

Supplementary Table S1: **Comparison at ART initiation of South African patients excluded from and included in the final analysis based on cohort-level ability to link to the National Population Register**

|  | **Excluded*** | | **Included**** | | **Total***** | |
| --- | --- | --- | --- | --- | --- | --- |
| *Patients* | 17 657 | | 30 467 | | 48 124 | |
| *Cohorts* | 4 | | 4 | | 8 | |
|  |  |  |  |  |  |  |
| **Gender, n(%)** | | | | | | |
| female | 11 123 | (63.0%) | 20 306 | (66.6%) | 31 429 | (65.3%) |
|  |  |  |  |  |  |  |
| **Age (years)** | | | | | | |
| median (IQR) | 35 | (30, 42) | 35 | (30, 41) | 35 | (30, 42) |
|  |  |  |  |  |  |  |
| **CD4 (cells/µl)** | | | | | | |
| observations, n(%) | 14 652 | (83.0%) | 27 211 | (89.3%) | 41 863 | (87.0%) |
| <25 | 2 073 | (14.1%) | 4 417 | (16.2%) | 6 490 | (15.5%) |
| 25-49 | 1 543 | (10.5%) | 3 257 | (12.0%) | 4 800 | (11.5%) |
| 50-99 | 2 718 | (18.6%) | 5 666 | (20.8%) | 8 384 | (20.0%) |
| 100-199 | 5 313 | (36.3%) | 10 853 | (39.9%) | 16 166 | (38.6%) |
| 200-349 | 2 163 | (14.8%) | 2 466 | (9.1%) | 4 629 | (11.1%) |
| 350-499 | 500 | (3.4%) | 325 | (1.2%) | 825 | (2.0%) |
| >=500 | 342 | (2.3%) | 227 | (0.8%) | 569 | (1.4%) |
|  |  |  |  |  |  |  |
| median (IQR) | 117 | (50, 187) | 102 | (42, 166) | 107 | (45, 172) |
|  |  |  |  |  |  |  |
| **Viral load (log_10_ copies/mL)** | | | | | | |
| observations, n(%) | 13 284 | (75.2%) | 12 129 | (39.8%) | 25 413 | (52.8%) |
| < 4 | 3 024 | (22.8%) | 3 198 | (26.4%) | 6 222 | (24.5%) |
| 4 to 5 | 5 056 | (38.1%) | 5 331 | (44.0%) | 10 387 | (40.9%) |
| >5 | 5 204 | (39.2%) | 3 600 | (29.7%) | 8 804 | (34.6%) |
|  |  |  |  |  |  |  |
| median (IQR) | 4.8 | (4.1, 5.3) | 4.6 | (3.9, 5.2) | 4.68 | (4.0, 5.2) |
|  |  |  |  |  |  |  |
| **Year of ART initiation** | | | | | | |
| 2001-2003 | 287 | (1.6%) | 1 022 | (3.4%) | 1 309 | (2.7%) |
| 2004-2006 | 9 052 | (51.3%) | 16 221 | (53.2%) | 25 273 | (52.5%) |
| 2007-2009 | 8 318 | (47.1%) | 13 224 | (43.4%) | 21 542 | (44.8%) |
|  |  |  |  |  |  |  |
| **Clinical stage, n(%)** |  |  |  |  |  |  |
| observations | 4 256 | (24.1%) | 12 857 | (42.2%) | 17 113 | (35.6%) |
| advanced | 3 227 | (38.8%) | 10 278 | (77.7%) | 13 505 | (62.7%) |
|  |  |  |  |  |  |  |
| IQR - inter-quartile range; advanced stage refers to WHO stage IV or CDC Stage C | | | | | |  |
| * Gugulethu, Tygerberg Hospital, Masiphumelele and Aurum cohorts | | | | |  |  |
| ** Khayelitsha, Thembalethu, McCord and Hlabisa cohorts | | | |  |  |  |
| *** p-values based on chi-squared tests for categorical comparisons and ranksum test for comparisons of continuous variables were <0.001 for all characteristics as a result of the large numbers of patients being compared | | | | | | |
