# Supplementary material for: Mortality in Patients with HIV-1 Infection Starting Antiretroviral Therapy in South Africa, Europe, or North America: A Collaborative Analysis of Prospective Studies
Source: PLoS Med. 2014 Sep 9;11(9):e1001718. doi: 10.1371/journal.pmed.1001718 (PMC4159124; doi:10.1371/journal.pmed.1001718)
Supplement: Table S2 — Comparison at ART initiation of patients lost to follow-up in South Africa with and without civil identifiers enabling linkage to the population register. (DOCX) [file pmed.1001718.s004.docx]

Supplementary Table S2: **Comparison at ART initiation of patients lost to follow-up in South Africa with and without civil identifiers enabling linkage to the population register**

| *Civil identifier present* | **No ID** | | **With ID** | | **p-value*** | **Total** | |
| --- | --- | --- | --- | --- | --- | --- | --- |
| *Patients included* | 1 773 | | 2 594 | |  | 4 367 | |
|  |  |  |  |  |  |  |  |
| **Gender, n(%)** |  |  |  |  |  |  |  |
| female | 1 130 | (63.7%) | 1 596 | (61.5%) | 0.139 | 2 726 | (62.4%) |
|  |  |  |  |  |  |  |  |
| **Age (years)** |  |  |  |  |  |  |  |
| median (IQR) | 34 | (29, 41) | 34 | (29, 40) | 0.127 | 34 | (29, 41) |
|  |  |  |  |  |  |  |  |
| **CD4 (cells/µl)** |  |  |  |  | 0.553 |  |  |
| observations, n(%) | 1 545 | (87.1%) | 2 305 | (88.9%) |  | 3 850 | (88.2%) |
| <25 | 303 | (19.6%) | 437 | (19.0%) |  | 740 | (19.2%) |
| 25-49 | 202 | (13.1%) | 312 | (13.5%) |  | 514 | (13.4%) |
| 50-99 | 298 | (19.3%) | 499 | (21.6%) |  | 797 | (20.7%) |
| 100-199 | 560 | (36.2%) | 815 | (35.4%) |  | 1 375 | (35.7%) |
| 200-349 | 150 | (9.7%) | 202 | (8.8%) |  | 352 | (9.1%) |
| 350-499 | 20 | (1.3%) | 22 | (1.0%) |  | 42 | (1.1%) |
| >=500 | 12 | (0.8%) | 18 | (0.8%) |  | 30 | (0.8%) |
| median (IQR) | 94 | (35, 161) | 88 | (36, 158) | 0.504 | 90 | (35, 159) |
|  |  |  |  |  |  |  |  |
| **Viral load (log_10_ copies/mL)** | |  |  |  | 0.106 |  |  |
| observations, n(%) | 574 | (32.4%) | 882 | (34.0%) |  | 1 456 | (33.3%) |
| < 4 | 123 | (21.4%) | 219 | (24.8%) |  | 342 | (23.5%) |
| 4 to 5 | 304 | (53.0%) | 418 | (47.4%) |  | 722 | (49.6%) |
| >5 | 147 | (25.6%) | 245 | (27.8%) |  | 392 | (26.9%) |
| median (IQR) | 4.5 | (4.1, 5.0) | 4.6 | (4.0, 5.1) | 0.788 | 4.6 | (4.0, 5.1) |
|  |  |  |  |  |  |  |  |
| **Year of ART initiation** |  |  |  |  | <0.001 |  |  |
| 2001-2003 | 69 | (3.9%) | 34 | (1.3%) |  | 103 | (2.4%) |
| 2004-2006 | 1 203 | (67.9%) | 1 583 | (61.0%) |  | 2 786 | (63.8%) |
| 2007-2009 | 501 | (28.3%) | 977 | (37.7%) |  | 1 478 | (33.8%) |
|  |  |  |  |  |  |  |  |
| **Clinical stage, n(%)** |  |  |  |  |  |  |  |
| observations | 450 | (25.4%) | 773 | (29.8%) |  | 1 223 | (28.0%) |
| advanced | 382 | (84.9%) | 672 | (86.9%) | 0.318 | 1 054 | (86.2%) |
|  |  |  |  |  |  |  |  |
| IQR - inter-quartile range; advanced stage refers to WHO stage IV or CDC Stage C | | | | | | |  |
| ID - civil identification number enabling linkage with death registry | | | | | |  |  |
| * chi-squared test for categorical comparisons and ranksum test for comparisons of continuous variables | | | | | | | |
