# Supplementary material for: Mortality in Patients with HIV-1 Infection Starting Antiretroviral Therapy in South Africa, Europe, or North America: A Collaborative Analysis of Prospective Studies
Source: PLoS Med. 2014 Sep 9;11(9):e1001718. doi: 10.1371/journal.pmed.1001718 (PMC4159124; doi:10.1371/journal.pmed.1001718)
Supplement: Textbox S1 — Multiple Imputation of missing baseline values. (DOCX) [file pmed.1001718.s005.docx]

**Supplementary Textbox S1: Multiple Imputation of missing baseline values**

Missing values of log viral load, CD4 count, and clinical stage were multiply imputed based on draws from the predictive posterior distribution of the missing data given the observed data. The analysis model (piecewise exponential survival model) was fitted on each imputed dataset, with results combined by means of “Rubin’s rules”.[1] Since the missing data relate mostly to administrative and clerical errors, possibly varying in different treatment centres, the missing at random assumption (that probability distribution of missing values depends only on observed quantities) needed for multiple imputation is likely to hold.

The relevant imputation draws were generated by specifying individual conditional distributions for each variable given other variables (fully conditional modelling) and iteratively drawing and updating imputed values from these distributions. Such “multiple imputation by chained equations” was implemented using Stata’s *ice* command.[2] We created 5 multiply imputed datasets. Our imputation model contained the following variables: cohort, gender, age, log viral load, year, outcome event, follow-up time, WHO/CDC stage, and region. Individual regressions fitted to predict the missing values of log viral load, CD4 count, and clinical stage were based on linear and logistic models for continuous and binary variables respectively.

The option “match” of Stata’s *ice* command was applied to CD4 and log viral load to ensure that imputed values only relate to values that exist in the real dataset. Under this option the closest observed value to the suggested imputation value is chosen so that, for example negative imputed values are not possible if all observed values are positive. Imputations were stratified by region to guarantee that the imputation models are based only on associations within regions.

The algorithm converged successfully. The analyses (piecewise exponential survival models) fitted to the imputed data were combined using Rubin’s rules by means of Stata’s *mi* estimate command.

References

1. Rubin DB (1996) Multiple Imputation After 18+ Years. Journal of the American Statistical Association 91: 473-489.

2. White IR, Royston P, Wood AM (2011) Multiple imputation using chained equations: Issues and guidance for practice. Statistics in Medicine 30: 377-399.
